# Dichlorido(4,7-diphenyl-1,10-phenanthroline-κ^2^ *N*,*N*′)gold(III) tetra­chloridoaurate(III)

**DOI:** 10.1107/S1600536808025476

**Published:** 2008-08-13

**Authors:** Roya Ahmadi, Vahid Amani, Hamid Reza Khavasi

**Affiliations:** a Islamic Azad University, Shahr-e-Rey Branch, Tehran, Iran; b Department of Chemistry, Shahid Beheshti University, Tehran 1983963113, Iran

## Abstract

In the cation of the title compound, [AuCl_2_(C_24_H_16_N_2_)][AuCl_4_], the Au^III^ atom is four-coordinated in a distorted square-planar configuration by two N atoms from a 4,7-diphenyl-1,10-phenanthroline ligand and two terminal Cl atoms. In the anion, the Au^III^ atom has a square-planar coordination. In the crystal structure, intra- and inter­molecular C—H⋯Cl hydrogen bonds are found.

## Related literature

For related literature, see: Hojjat Kashani *et al.* (2008 ▶); Mclnnes *et al.* (1995 ▶); Bjernemose *et al.* (2004 ▶); Hayoun *et al.* (2006 ▶); Abbate *et al.* (2000 ▶); Adams & Strahle (1982 ▶).
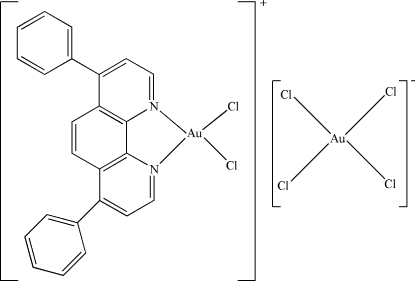


## Experimental

### 

#### Crystal data

[AuCl_2_(C_24_H_16_N_2_)][AuCl_4_]
*M* *_r_* = 939.03
Monoclinic, 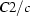

*a* = 26.2625 (16) Å
*b* = 13.7608 (6) Å
*c* = 14.4292 (9) Å
β = 101.207 (5)°
*V* = 5115.2 (5) Å^3^
*Z* = 8
Mo *K*α radiation
μ = 12.10 mm^−1^
*T* = 120 (2) K
0.43 × 0.35 × 0.30 mm

#### Data collection

Bruker SMART CCD area-detector diffractometer
Absorption correction: numerical (*X-SHAPE* and *X-RED*; Stoe & Cie, 2005 ▶) *T*_min_ = 0.580, *T*_max_ = 0.640
18667 measured reflections
6864 independent reflections
6404 reflections with *I* > 2σ(*I*)
*R*_int_ = 0.090

#### Refinement

*R*[*F* ^2^ > 2σ(*F* ^2^)] = 0.068
*wR*(*F* ^2^) = 0.173
*S* = 1.16
6864 reflections
308 parameters
H-atom parameters constrained
Δρ_max_ = 1.07 e Å^−3^
Δρ_min_ = −1.02 e Å^−3^

### 

Data collection: *SMART* (Bruker, 1998 ▶); cell refinement: *SMART*; data reduction: *SAINT* (Bruker, 1998 ▶); program(s) used to solve structure: *SHELXTL* (Sheldrick, 2008 ▶); program(s) used to refine structure: *SHELXTL*; molecular graphics: *ORTEP-3 for Windows* (Farrugia, 1997 ▶); software used to prepare material for publication: *WinGX* (Farrugia, 1999 ▶).

## Supplementary Material

Crystal structure: contains datablocks I, global. DOI: 10.1107/S1600536808025476/hk2509sup1.cif

Structure factors: contains datablocks I. DOI: 10.1107/S1600536808025476/hk2509Isup2.hkl

Additional supplementary materials:  crystallographic information; 3D view; checkCIF report

## Figures and Tables

**Table:** 

Au1—N2	2.032 (6)
Au1—N1	2.039 (7)
Au1—Cl2	2.2546 (19)
Au1—Cl1	2.257 (2)
Au2—Cl4	2.281 (2)
Au2—Cl5	2.281 (2)
Au2—Cl6	2.284 (2)
Au2—Cl3	2.285 (2)

**Table:** 

N2—Au1—N1	81.1 (3)
N2—Au1—Cl2	175.42 (19)
N1—Au1—Cl2	94.3 (2)
N2—Au1—Cl1	94.92 (19)
N1—Au1—Cl1	175.95 (19)
Cl2—Au1—Cl1	89.62 (8)
Cl4—Au2—Cl5	90.26 (10)
Cl4—Au2—Cl6	178.77 (8)
Cl5—Au2—Cl6	89.67 (9)
Cl4—Au2—Cl3	89.96 (10)
Cl5—Au2—Cl3	178.75 (7)
Cl6—Au2—Cl3	90.14 (9)

**Table 2:** Hydrogen-bond geometry (Å, °)

*D*—H⋯*A*	*D*—H	H⋯*A*	*D*⋯*A*	*D*—H⋯*A*
C1—H1⋯Cl2	0.93	2.68	3.244 (9)	120
C1—H1⋯Cl6^i^	0.93	2.79	3.668 (8)	159
C18—H18⋯Cl2^ii^	0.93	2.79	3.653 (8)	155
C22—H22⋯Cl1	0.93	2.66	3.239 (8)	121
C22—H22⋯Cl4^iii^	0.93	2.76	3.555 (9)	143

Symmetry codes: (i) 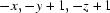; (ii) 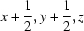; (iii) 
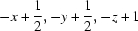
.

**Fig. 1.**
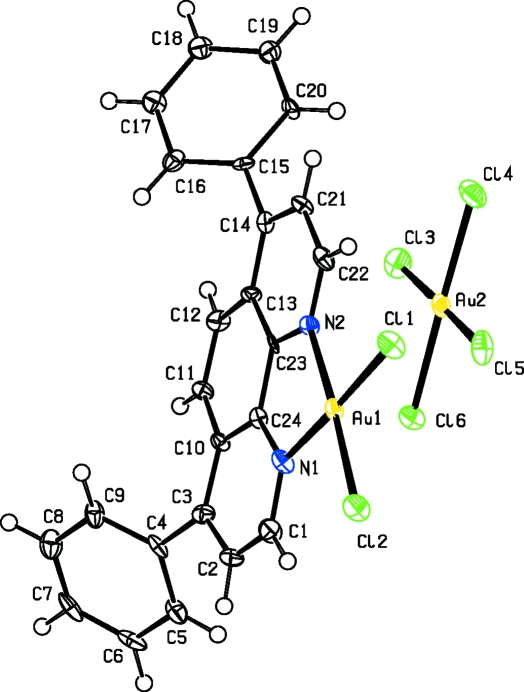
The molecular structure of the title molecule, with the atom-numbering scheme. Displacement ellipsoids are drawn at the 50% probability level.

## supplementary crystallographic information

### Comment

Recently, we reported the synthesis and crystal structure of [H_2_DA18C6]- [AuCl_4_].2H_2_O, (II) (Hojjat Kashani *et al.*, 2008) [where H_2_DA18C6 is 1,10-Diazonia-18-crown-6]. There are several Au^III^ complexes, with formula, [AuCl_2_(N—N)], such as [AuCl_2_(bipy)][BF_4_], (III) (Mclnnes *et al.*, 1995), [AuCl_2_(bipy)](NO_3_), (IV) (Bjernemose *et al.*,  2004), [AuCl_2_(bipy)]- [AuBr_4_], (V) (Hayoun *et al.*,  2006) and [AuCl_2_(phen)]Cl.H_2_O, (VI) (Abbate *et al.*,  2000) [where bipy is 2,2'-bipyridine and phen is 1,10-phenanthroline] have been synthesized and characterized by single-crystal X-ray diffraction methods. There are also two Au^III^ complexes, with formula, [AuCl_2_L_2_], such as [AuCl_2_(py)_2_][AuCl_4_], (VII) and [AuCl_2_(py)_2_]Cl.H_2_O, (VIII) (Adams & Strahle, 1982) [where py is pyridine] have been synthesized and characterized by single-crystal X-ray diffraction methods. We report herein the synthesis and crystal structure of the title compound, (I).

The asymmetric unit of (I), (Fig. 1) contains one cation and one anion. In the cation, the Au^III^ atom is four-coordinated in a distorted square-planar configuration by two N atoms from 4,7-diphenyl-1,10-phenanthroline ligand and two terminal Cl atoms. In the anion, the Au ion has a square-planar coordination. In the cation, the Au-Cl and Au-N bond lengths and angles (Table 1) are in good agreement with the corresponding values in (III) and (IV). In the anion, the Au-Cl bond lengths and angles (Table 1) are within normal ranges.

In the crystal structure, intra- and intermolecular C-H···Cl hydrogen bonds (Table 2) link the molecules, in which they may be effective in the stabilization of the structure.

### Experimental

For the preparation of the title compound, a solution of 4,7-diphenyl-1,10- phenanthroline (0.21 g, 0.63 mmol) in EtOH (30 ml) was added to a solution of HAuCl_4_.3H_2_O, (0.25 g, 0.63 mmol) in acetonitrile (40 ml) and the resulting yellow solution was stirred for 10 min at 313 K. Then, it was left to evaporate slowly at room temperature. After one week, yellow prismatic crystals were isolated (yield; 0.45 g, 75.8%, m.p. < 573 K).

### Refinement

H atoms were positioned geometrically, with C-H = 0.93 Å for aromatic H, and constrained to ride on their parent atoms with U_iso_(H) = 1.2U_eq_(C).

### Crystal data

**Table:**

[AuCl_2_(C_24_H_16_N_2_)][AuCl_4_]	*F*_000_ = 3472
*M**_r_* = 939.03	*D*_x_ = 2.439 Mg m^−^^3^
Monoclinic, *C*2/*c*	Mo *K*α radiation λ = 0.71073 Å
Hall symbol: -C 2yc	Cell parameters from 2231 reflections
*a* = 26.2625 (16) Å	θ = 1.7–29.2º
*b* = 13.7608 (6) Å	µ = 12.10 mm^−^^1^
*c* = 14.4292 (9) Å	*T* = 120 (2) K
β = 101.207 (5)º	Prism, yellow
*V* = 5115.2 (5) Å^3^	0.43 × 0.35 × 0.30 mm
*Z* = 8	

### Data collection

**Table:**

Bruker SMART CCD area-detector diffractometer	6864 independent reflections
Radiation source: fine-focus sealed tube	6404 reflections with *I* > 2σ(*I*)
Monochromator: graphite	*R*_int_ = 0.090
*T* = 120(2) K	θ_max_ = 29.2º
φ and ω scans	θ_min_ = 1.7º
Absorption correction: numericalshape of crystal determined optically (PROGRAM? Reference?)	*h* = −35→35
*T*_min_ = 0.580, *T*_max_ = 0.640	*k* = −18→18
18667 measured reflections	*l* = −19→13

### Refinement

**Table:**

Refinement on *F*^2^	Hydrogen site location: inferred from neighbouring sites
Least-squares matrix: full	H-atom parameters constrained
*R*[*F*^2^ > 2σ(*F*^2^)] = 0.068	*w* = 1/[σ^2^(*F*_o_^2^) + (0.0977*P*)^2^ + 20.7667*P*] where *P* = (*F*_o_^2^ + 2*F*_c_^2^)/3
*wR*(*F*^2^) = 0.173	(Δ/σ)_max_ = 0.048
*S* = 1.16	Δρ_max_ = 1.07 e Å^−^^3^
6864 reflections	Δρ_min_ = −1.02 e Å^−^^3^
308 parameters	Extinction correction: SHELXTL (Sheldrick, 2008), Fc^*^=kFc[1+0.001xFc^2^λ^3^/sin(2θ)]^-1/4^
Primary atom site location: structure-invariant direct methods	Extinction coefficient: 0.00055 (6)
Secondary atom site location: difference Fourier map	

### Special details

**Table:**

Geometry. All e.s.d.'s (except the e.s.d. in the dihedral angle between two l.s. planes) are estimated using the full covariance matrix. The cell e.s.d.'s are taken into account individually in the estimation of e.s.d.'s in distances, angles and torsion angles; correlations between e.s.d.'s in cell parameters are only used when they are defined by crystal symmetry. An approximate (isotropic) treatment of cell e.s.d.'s is used for estimating e.s.d.'s involving l.s. planes.
Refinement. Refinement of F^2^ against ALL reflections. The weighted R-factor wR and goodness of fit S are based on F^2^, conventional R-factors R are based on F, with F set to zero for negative F^2^. The threshold expression of F^2^ > 2sigma(F^2^) is used only for calculating R-factors(gt) etc. and is not relevant to the choice of reflections for refinement. R-factors based on F^2^ are statistically about twice as large as those based on F, and R- factors based on ALL data will be even larger.

### Fractional atomic coordinates and isotropic or equivalent isotropic displacement parameters (Å^2^)

**Table:**

	*x*	*y*	*z*	*U*_iso_*/*U*_eq_	
Au1	0.097267 (11)	−0.152249 (19)	0.112685 (18)	0.01435 (13)	
Au2	0.165438 (12)	0.96986 (2)	0.854512 (18)	0.01739 (13)	
Cl1	0.12293 (8)	−0.30783 (14)	0.10295 (15)	0.0240 (4)	
Cl2	0.01813 (8)	−0.20496 (16)	0.12856 (16)	0.0259 (4)	
Cl3	0.21356 (9)	1.10394 (18)	0.83479 (15)	0.0280 (4)	
Cl4	0.23388 (10)	0.87252 (19)	0.84084 (16)	0.0320 (5)	
Cl5	0.11779 (10)	0.83657 (16)	0.87768 (15)	0.0284 (5)	
Cl6	0.09622 (8)	1.06689 (16)	0.86481 (15)	0.0257 (4)	
N1	0.0787 (3)	−0.0088 (5)	0.1182 (5)	0.0160 (12)	
N2	0.1667 (2)	−0.0939 (5)	0.1005 (4)	0.0138 (11)	
C1	0.0334 (3)	0.0290 (6)	0.1291 (6)	0.0203 (15)	
H1	0.0055	−0.0117	0.1321	0.024*	
C2	0.0279 (3)	0.1283 (7)	0.1359 (6)	0.0193 (14)	
H2	−0.0041	0.1535	0.1422	0.023*	
C3	0.0691 (3)	0.1922 (6)	0.1336 (5)	0.0159 (13)	
C4	0.0612 (3)	0.2989 (6)	0.1480 (5)	0.0161 (13)	
C5	0.0146 (4)	0.3441 (6)	0.1051 (6)	0.0211 (16)	
H5	−0.0114	0.3090	0.0661	0.025*	
C6	0.0082 (3)	0.4422 (7)	0.1221 (6)	0.0239 (16)	
H6	−0.0222	0.4734	0.0937	0.029*	
C7	0.0463 (4)	0.4939 (6)	0.1804 (6)	0.0229 (16)	
H7	0.0418	0.5599	0.1898	0.027*	
C8	0.0914 (4)	0.4485 (6)	0.2254 (6)	0.0243 (16)	
H8	0.1167	0.4834	0.2660	0.029*	
C9	0.0985 (4)	0.3503 (5)	0.2094 (6)	0.0194 (15)	
H9	0.1285	0.3191	0.2400	0.023*	
C10	0.1156 (3)	0.1527 (5)	0.1165 (5)	0.0127 (13)	
C11	0.1599 (3)	0.2079 (5)	0.1012 (5)	0.0146 (13)	
H11	0.1577	0.2754	0.0998	0.018*	
C12	0.2050 (3)	0.1647 (5)	0.0888 (6)	0.0161 (13)	
H12	0.2322	0.2029	0.0769	0.019*	
C13	0.2106 (3)	0.0604 (5)	0.0940 (5)	0.0125 (12)	
C14	0.2567 (3)	0.0094 (5)	0.0877 (5)	0.0139 (12)	
C15	0.3067 (3)	0.0582 (5)	0.0815 (5)	0.0133 (12)	
C16	0.3258 (3)	0.1344 (6)	0.1443 (5)	0.0178 (14)	
H16	0.3066	0.1577	0.1874	0.021*	
C17	0.3743 (3)	0.1738 (6)	0.1401 (6)	0.0194 (14)	
H17	0.3868	0.2254	0.1796	0.023*	
C18	0.4039 (3)	0.1381 (6)	0.0787 (6)	0.0202 (15)	
H18	0.4366	0.1637	0.0782	0.024*	
C19	0.3838 (3)	0.0621 (6)	0.0167 (5)	0.0195 (14)	
H19	0.4032	0.0384	−0.0258	0.023*	
C20	0.3364 (3)	0.0230 (5)	0.0182 (5)	0.0146 (13)	
H20	0.3236	−0.0272	−0.0230	0.018*	
C21	0.2552 (3)	−0.0924 (6)	0.0870 (5)	0.0160 (13)	
H21	0.2849	−0.1274	0.0823	0.019*	
C22	0.2093 (3)	−0.1420 (6)	0.0935 (5)	0.0170 (14)	
H22	0.2089	−0.2096	0.0929	0.020*	
C23	0.1672 (3)	0.0044 (5)	0.1023 (4)	0.0129 (13)	
C24	0.1190 (3)	0.0514 (5)	0.1122 (5)	0.0113 (12)	

### Atomic displacement parameters (Å^2^)

**Table:**

	*U*^11^	*U*^22^	*U*^33^	*U*^12^	*U*^13^	*U*^23^
Au1	0.01838 (19)	0.01279 (17)	0.01316 (17)	−0.00363 (8)	0.00622 (12)	−0.00142 (9)
Au2	0.0225 (2)	0.01961 (19)	0.00978 (17)	0.00323 (9)	0.00248 (12)	−0.00040 (9)
Cl1	0.0312 (9)	0.0133 (8)	0.0308 (9)	−0.0030 (7)	0.0144 (8)	0.0002 (7)
Cl2	0.0258 (9)	0.0216 (9)	0.0340 (10)	−0.0098 (7)	0.0152 (8)	−0.0046 (8)
Cl3	0.0315 (10)	0.0319 (11)	0.0216 (8)	−0.0063 (8)	0.0079 (8)	0.0042 (8)
Cl4	0.0382 (12)	0.0347 (11)	0.0229 (9)	0.0178 (10)	0.0057 (9)	−0.0016 (9)
Cl5	0.0424 (12)	0.0248 (10)	0.0176 (8)	−0.0076 (8)	0.0050 (8)	−0.0014 (7)
Cl6	0.0248 (9)	0.0259 (10)	0.0272 (9)	0.0051 (7)	0.0066 (8)	−0.0032 (8)
N1	0.023 (3)	0.012 (3)	0.016 (3)	−0.002 (2)	0.010 (2)	0.002 (2)
N2	0.015 (3)	0.016 (3)	0.013 (2)	−0.003 (2)	0.007 (2)	0.001 (2)
C1	0.022 (4)	0.017 (4)	0.024 (4)	−0.005 (3)	0.009 (3)	−0.003 (3)
C2	0.012 (3)	0.026 (4)	0.022 (3)	0.000 (3)	0.008 (3)	−0.003 (3)
C3	0.015 (3)	0.016 (3)	0.016 (3)	0.003 (3)	0.001 (3)	0.004 (3)
C4	0.024 (3)	0.014 (3)	0.014 (3)	0.004 (3)	0.011 (3)	−0.002 (3)
C5	0.027 (4)	0.018 (4)	0.018 (3)	0.005 (3)	0.007 (3)	−0.003 (3)
C6	0.025 (4)	0.027 (4)	0.023 (4)	0.014 (3)	0.015 (3)	0.004 (3)
C7	0.038 (5)	0.016 (3)	0.021 (3)	0.001 (3)	0.022 (3)	−0.002 (3)
C8	0.031 (4)	0.016 (3)	0.025 (4)	−0.003 (3)	0.003 (3)	−0.004 (3)
C9	0.030 (4)	0.012 (3)	0.016 (3)	0.002 (3)	0.002 (3)	−0.001 (3)
C10	0.014 (3)	0.014 (3)	0.012 (3)	−0.002 (2)	0.007 (2)	0.000 (2)
C11	0.016 (3)	0.015 (3)	0.015 (3)	0.002 (2)	0.006 (3)	0.002 (3)
C12	0.015 (3)	0.012 (3)	0.022 (3)	−0.006 (2)	0.006 (3)	0.002 (3)
C13	0.009 (3)	0.015 (3)	0.015 (3)	−0.001 (2)	0.006 (2)	0.000 (3)
C14	0.018 (3)	0.012 (3)	0.012 (3)	−0.002 (2)	0.003 (3)	−0.001 (2)
C15	0.007 (3)	0.017 (3)	0.015 (3)	0.003 (2)	0.001 (2)	0.003 (3)
C16	0.016 (3)	0.018 (3)	0.017 (3)	0.002 (3)	−0.001 (3)	0.002 (3)
C17	0.021 (4)	0.015 (3)	0.022 (3)	−0.001 (3)	0.006 (3)	0.000 (3)
C18	0.019 (4)	0.016 (3)	0.026 (4)	−0.001 (3)	0.005 (3)	−0.001 (3)
C19	0.019 (3)	0.024 (4)	0.016 (3)	−0.001 (3)	0.005 (3)	−0.001 (3)
C20	0.018 (3)	0.018 (3)	0.010 (3)	−0.001 (2)	0.008 (3)	−0.001 (2)
C21	0.017 (3)	0.016 (3)	0.018 (3)	0.002 (2)	0.009 (3)	−0.001 (3)
C22	0.024 (4)	0.016 (3)	0.013 (3)	0.004 (3)	0.008 (3)	0.002 (3)
C23	0.018 (3)	0.017 (3)	0.005 (3)	0.003 (3)	0.004 (2)	−0.004 (3)
C24	0.015 (3)	0.009 (3)	0.011 (3)	−0.006 (2)	0.007 (2)	0.001 (2)

### Geometric parameters (Å, °)

**Table:**

Au1—N2	2.032 (6)	C10—C11	1.442 (10)
Au1—N1	2.039 (7)	C11—C12	1.367 (10)
Au1—Cl2	2.2546 (19)	C11—H11	0.9300
Au1—Cl1	2.257 (2)	C12—C13	1.444 (10)
Au2—Cl4	2.281 (2)	C12—H12	0.9300
Au2—Cl5	2.281 (2)	C13—C23	1.401 (9)
Au2—Cl6	2.284 (2)	C13—C14	1.417 (10)
Au2—Cl3	2.285 (2)	C14—C21	1.400 (10)
C1—N1	1.335 (10)	C14—C15	1.494 (10)
C1—C2	1.379 (11)	C15—C20	1.398 (9)
C1—H1	0.9300	C15—C16	1.412 (11)
C2—C3	1.399 (10)	C16—C17	1.395 (11)
C2—H2	0.9300	C16—H16	0.9300
C3—C10	1.403 (10)	C17—C18	1.378 (12)
C3—C4	1.502 (10)	C17—H17	0.9300
C4—C9	1.381 (11)	C18—C19	1.411 (11)
C4—C5	1.404 (11)	C18—H18	0.9300
C5—C6	1.389 (11)	C19—C20	1.361 (10)
C5—H5	0.9300	C19—H19	0.9300
C6—C7	1.373 (14)	C20—H20	0.9300
C6—H6	0.9300	C21—C22	1.405 (11)
C7—C8	1.383 (13)	C21—H21	0.9300
C7—H7	0.9300	C22—N2	1.320 (10)
C8—C9	1.390 (11)	C22—H22	0.9300
C8—H8	0.9300	C23—N2	1.352 (9)
C9—H9	0.9300	C23—C24	1.454 (9)
C10—C24	1.399 (9)	C24—N1	1.359 (9)
			
N2—Au1—N1	81.1 (3)	C11—C12—C13	120.6 (7)
N2—Au1—Cl2	175.42 (19)	C11—C12—H12	119.7
N1—Au1—Cl2	94.3 (2)	C13—C12—H12	119.7
N2—Au1—Cl1	94.92 (19)	C23—C13—C14	116.9 (7)
N1—Au1—Cl1	175.95 (19)	C23—C13—C12	118.3 (6)
Cl2—Au1—Cl1	89.62 (8)	C14—C13—C12	124.8 (6)
Cl4—Au2—Cl5	90.26 (10)	C21—C14—C13	118.2 (7)
Cl4—Au2—Cl6	178.77 (8)	C21—C14—C15	118.3 (7)
Cl5—Au2—Cl6	89.67 (9)	C13—C14—C15	123.5 (7)
Cl4—Au2—Cl3	89.96 (10)	C20—C15—C16	120.2 (7)
Cl5—Au2—Cl3	178.75 (7)	C20—C15—C14	119.4 (7)
Cl6—Au2—Cl3	90.14 (9)	C16—C15—C14	120.2 (6)
N1—C1—C2	120.1 (7)	C17—C16—C15	118.3 (7)
N1—C1—H1	119.9	C17—C16—H16	120.8
C2—C1—H1	119.9	C15—C16—H16	120.9
C1—C2—C3	121.9 (7)	C18—C17—C16	121.5 (8)
C1—C2—H2	119.0	C18—C17—H17	119.2
C3—C2—H2	119.0	C16—C17—H17	119.2
C2—C3—C10	117.7 (7)	C17—C18—C19	118.9 (8)
C2—C3—C4	118.9 (7)	C17—C18—H18	120.6
C10—C3—C4	123.5 (7)	C19—C18—H18	120.5
C9—C4—C5	120.3 (7)	C20—C19—C18	120.9 (7)
C9—C4—C3	119.3 (7)	C20—C19—H19	119.5
C5—C4—C3	120.2 (7)	C18—C19—H19	119.6
C6—C5—C4	118.5 (8)	C19—C20—C15	120.1 (7)
C6—C5—H5	120.7	C19—C20—H20	119.9
C4—C5—H5	120.8	C15—C20—H20	120.0
C7—C6—C5	120.9 (8)	C14—C21—C22	120.7 (7)
C7—C6—H6	119.6	C14—C21—H21	119.6
C5—C6—H6	119.5	C22—C21—H21	119.7
C6—C7—C8	120.6 (8)	N2—C22—C21	120.7 (7)
C6—C7—H7	119.7	N2—C22—H22	119.7
C8—C7—H7	119.7	C21—C22—H22	119.6
C7—C8—C9	119.4 (8)	N2—C23—C13	123.6 (6)
C7—C8—H8	120.3	N2—C23—C24	116.2 (6)
C9—C8—H8	120.3	C13—C23—C24	120.2 (6)
C4—C9—C8	120.2 (8)	N1—C24—C10	123.2 (7)
C4—C9—H9	119.9	N1—C24—C23	116.0 (6)
C8—C9—H9	119.9	C10—C24—C23	120.8 (6)
C24—C10—C3	117.3 (7)	C1—N1—C24	119.5 (7)
C24—C10—C11	117.3 (6)	C1—N1—Au1	127.4 (5)
C3—C10—C11	125.4 (7)	C24—N1—Au1	113.1 (5)
C12—C11—C10	122.4 (7)	C22—N2—C23	119.9 (6)
C12—C11—H11	118.8	C22—N2—Au1	126.6 (5)
C10—C11—H11	118.8	C23—N2—Au1	113.5 (5)
			
N1—C1—C2—C3	−1.3 (13)	C18—C19—C20—C15	0.2 (12)
C1—C2—C3—C10	5.0 (12)	C16—C15—C20—C19	0.0 (11)
C1—C2—C3—C4	−175.9 (8)	C14—C15—C20—C19	−175.1 (7)
C2—C3—C4—C9	133.5 (8)	C13—C14—C21—C22	−0.7 (10)
C10—C3—C4—C9	−47.4 (10)	C15—C14—C21—C22	179.4 (7)
C2—C3—C4—C5	−41.7 (10)	C14—C21—C22—N2	−0.1 (11)
C10—C3—C4—C5	137.4 (8)	C14—C13—C23—N2	−2.9 (10)
C9—C4—C5—C6	3.2 (12)	C12—C13—C23—N2	174.8 (7)
C3—C4—C5—C6	178.3 (7)	C14—C13—C23—C24	178.0 (6)
C4—C5—C6—C7	−0.7 (12)	C12—C13—C23—C24	−4.3 (10)
C5—C6—C7—C8	−1.6 (13)	C3—C10—C24—N1	3.9 (10)
C6—C7—C8—C9	1.5 (13)	C11—C10—C24—N1	−175.0 (6)
C5—C4—C9—C8	−3.3 (12)	C3—C10—C24—C23	−175.0 (6)
C3—C4—C9—C8	−178.5 (8)	C11—C10—C24—C23	6.1 (10)
C7—C8—C9—C4	0.9 (13)	N2—C23—C24—N1	−0.2 (9)
C2—C3—C10—C24	−6.1 (10)	C13—C23—C24—N1	179.0 (6)
C4—C3—C10—C24	174.8 (7)	N2—C23—C24—C10	178.9 (6)
C2—C3—C10—C11	172.7 (7)	C13—C23—C24—C10	−2.0 (10)
C4—C3—C10—C11	−6.4 (11)	C2—C1—N1—C24	−1.2 (12)
C24—C10—C11—C12	−4.0 (11)	C2—C1—N1—Au1	176.9 (6)
C3—C10—C11—C12	177.2 (7)	C10—C24—N1—C1	−0.2 (11)
C10—C11—C12—C13	−2.3 (11)	C23—C24—N1—C1	178.8 (7)
C11—C12—C13—C23	6.5 (11)	C10—C24—N1—Au1	−178.6 (5)
C11—C12—C13—C14	−176.1 (7)	C23—C24—N1—Au1	0.4 (7)
C23—C13—C14—C21	2.1 (10)	N2—Au1—N1—C1	−178.6 (7)
C12—C13—C14—C21	−175.4 (7)	Cl2—Au1—N1—C1	0.7 (7)
C23—C13—C14—C15	−178.1 (6)	N2—Au1—N1—C24	−0.4 (5)
C12—C13—C14—C15	4.4 (11)	Cl2—Au1—N1—C24	179.0 (5)
C21—C14—C15—C20	42.6 (10)	C21—C22—N2—C23	−0.6 (10)
C13—C14—C15—C20	−137.3 (7)	C21—C22—N2—Au1	−178.9 (5)
C21—C14—C15—C16	−132.5 (7)	C13—C23—N2—C22	2.2 (10)
C13—C14—C15—C16	47.7 (10)	C24—C23—N2—C22	−178.7 (6)
C20—C15—C16—C17	1.0 (11)	C13—C23—N2—Au1	−179.3 (5)
C14—C15—C16—C17	176.0 (7)	C24—C23—N2—Au1	−0.2 (7)
C15—C16—C17—C18	−2.1 (12)	N1—Au1—N2—C22	178.7 (6)
C16—C17—C18—C19	2.2 (13)	N1—Au1—N2—C23	0.3 (5)
C17—C18—C19—C20	−1.2 (12)	Cl1—Au1—N2—C23	179.4 (4)

### Hydrogen-bond geometry (Å, °)

**Table:**

*D*—H···*A*	*D*—H	H···*A*	*D*···*A*	*D*—H···*A*
C1—H1···Cl2	0.93	2.68	3.244 (9)	120
C1—H1···Cl6^i^	0.93	2.79	3.668 (8)	159
C18—H18···Cl2^ii^	0.93	2.79	3.653 (8)	155
C22—H22···Cl1	0.93	2.66	3.239 (8)	121
C22—H22···Cl4^iii^	0.93	2.76	3.555 (9)	143

Symmetry codes: (i) −*x*, −*y*+1, −*z*+1; (ii) *x*+1/2, *y*+1/2, *z*; (iii) −*x*+1/2, −*y*+1/2, −*z*+1.
